# Prolonged survival of 3 dogs with *Prototheca bovis* infection treated with posaconazole

**DOI:** 10.1093/jvimsj/aalag170

**Published:** 2026-08-24

**Authors:** Kathrin F A Langner, Ammie Higgins, Margie Roser, Troy A Bunn, Amy Lam, Angelika Proskurnicka, Tomasz Jagielski, Richard Malik

**Affiliations:** Western Australian Veterinary Emergency and Specialty, Success, WA 6164, Australia; Pathwest Laboratory, Nedlands, WA 6009, Australia; Vetnostics, Macquarie Park, NSW 2113, Australia; Western Australian Veterinary Emergency and Specialty, Success, WA 6164, Australia; Small Animal Specialist Hospital, North Ryde, NSW 2113, Australia; Department of Medical Microbiology, Institute of Microbiology, Faculty of Biology, University of Warsaw, Warsaw 02-096, Poland; Department of Medical Microbiology, Institute of Microbiology, Faculty of Biology, University of Warsaw, Warsaw 02-096, Poland; Centre for Veterinary Education, Sydney School of Veterinary Science, The University of Sydney, Sydney 2050, Australia

**Keywords:** algal infection, antifungal treatment, canine protothecosis, enteric protothecosis, *Prototheca bovis*, posaconazole

## Abstract

Primary enteric or disseminated infections with *Prototheca bovis* in dogs typically are associated with short survival times despite treatment. We describe the extended clinical course of 3 dogs diagnosed with enteric *P. bovis* infection treated with antifungal therapy incorporating posaconazole. One dog was alive 45 months after starting treatment, exhibiting only mild, intermittent diarrhea and no detectable algae on rectal cytology. This dog also received SC amphotericin B (AMB) infusions during the first month after diagnosis. The second dog, managed exclusively with posaconazole, was alive after 39 months with moderate mixed-bowel diarrhea and ongoing *P. bovis* shedding but without clinical signs of dissemination. The third dog survived 21 months before death because of central nervous system dissemination; additional treatments included itraconazole, AMB, terbinafine, and immunotherapy using an autologous vaccine. Posaconazole was well tolerated in all dogs and can be considered a therapeutic option for dogs with enteric protothecosis.

## Introduction

Protothecosis is an emerging infection with achlorophyllic microalgae of the genus *Prototheca*.^1^ These algae are found in aqueous environments with high organic matter content and as contaminants in soil and food. Of 18 currently known *Prototheca* spp., *Prototheca bovis, Prototheca wickerhamii*, and *Prototheca ciferrii* have been identified as pathogens in dogs.^1^ Historically, *Prototheca zopfii* has been cited as the most common cause for protothecosis in dogs, but it was assumed that *P. bovis* and to a lesser degree *P. ciferrii* were the infecting species in these reports because many of the publications pre-dated taxonomic revision.^1–3^ Protothecosis in dogs most commonly occurs as primary colonization of the large bowel and subsequent invasion of the colonic wall.^1^^,^^2^ Over time, many dogs develop disseminated disease with involvement of disparate organ systems including the eyes, central nervous system (CNS), kidneys, heart, liver, and lungs after algal penetration of colonic venules.^1^^,^^2^ Tropism for the eyes and CNS is prominent. In a small number of dogs, the disease remains localized to the colon.^1^^,^^2^ Treatment involves empirical use of one or more antifungal drugs including various azoles and polyenes (amphotericin B [AMB] and nystatin).^1^^,^^2^ However, despite treatment, colonic and disseminated protothecosis ultimately is fatal in nearly all reported dogs.^1^^,^^2^ Our case series describes prolonged survival of 3 dogs diagnosed in 2022 with primary enteric protothecosis that received posaconazole either as the sole antifungal drug or as the main part of multimodal antifungal treatment.

## Case descriptions

### Case 1

**Figure 1:**
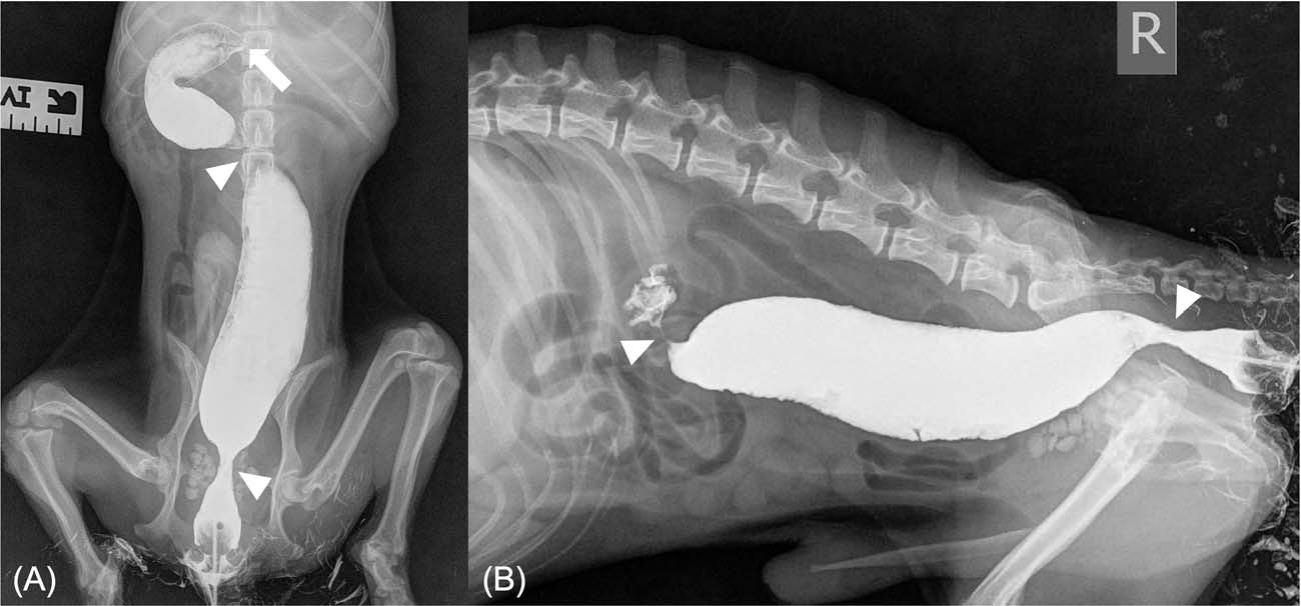
Colonogram of dog 1. (A) Ventro-dorsal and (B) right lateral abdominal projections were obtained immediately after instillation of 30% barium sulfate through a rectal Foley catheter. Mild rectal stenosis and moderate colonic stenosis (arrowheads) were noted. Additional colonic stenosis aboral to the ileocolic junction (arrow) was identified at exploratory laparotomy.

**Figure 2:**
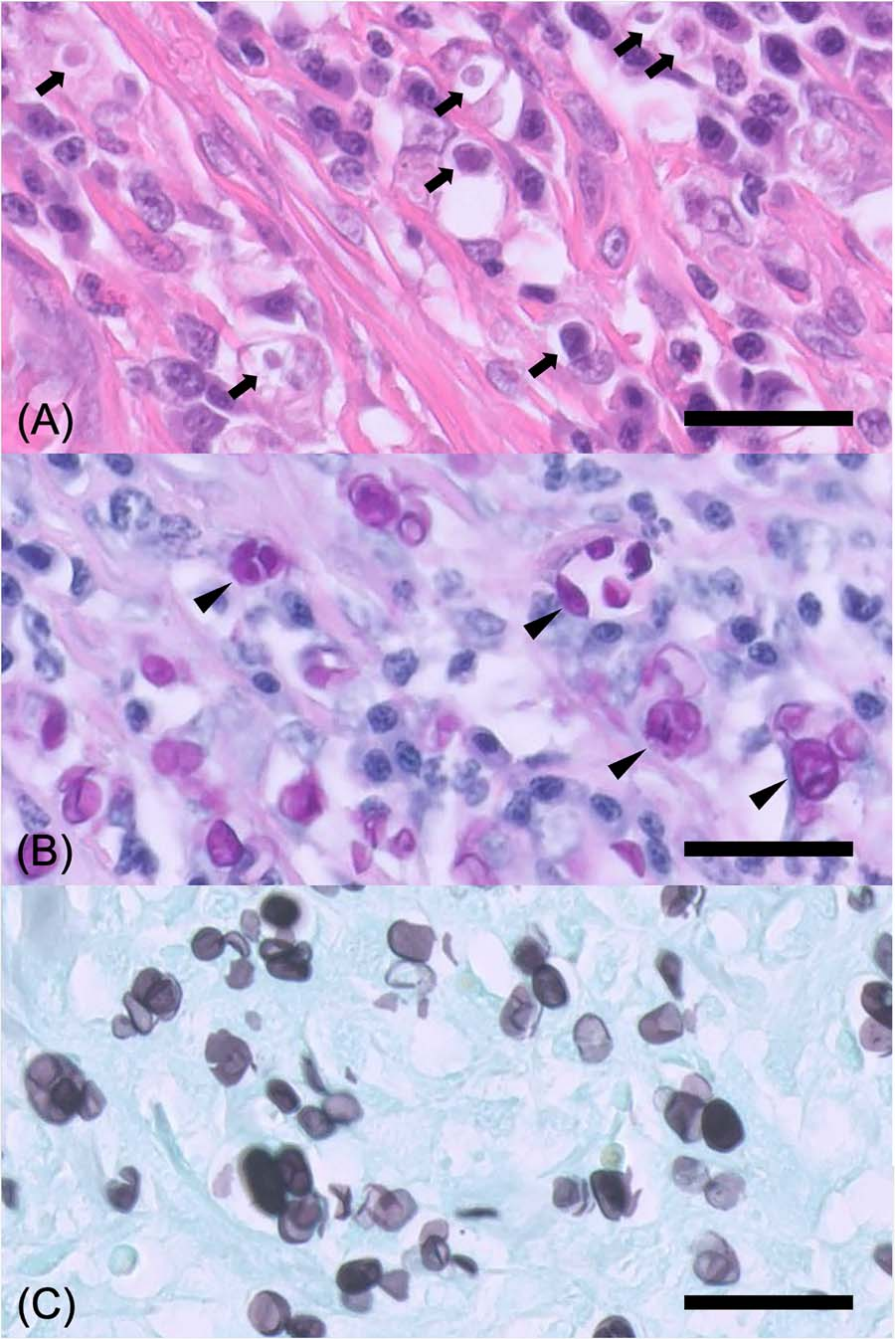
Histopathology of the colonic wall of dog 1. (A) Hematoxylin and eosin stain of submucosa show numerous algal organisms (arrows) extracellularly and within macrophages. (B) Algal organisms, often containing multiple endospores (arrowheads), stained dark with periodic acid-Schiff. (C) Algal organisms stained dark with Grocott’s methenamine silver stain. (A)-(C) 400× magnification; scale bar, 20 μm (images courtesy Dr Margie Roser, Vetnostics, North Ryde, Australia).

A 7-year-old intact male Maltese dog was referred with a 3-week history of progressive tenesmus, hematochezia, hyporexia, and 40% weight loss. Since adoption approximately 4 years ago, the dog had frequent mixed-bowel diarrhea. Upon presentation, a tense abdomen, low body condition score (BCS; 2/9) and severe perineal inflammation were observed. Hematology identified neutrophilia (27.8 × 10^9^/L; reference interval [RI]: 3-11.5) with left shift and moderate toxic changes, monocytosis (3.05 × 10^9^/L; RI: 0.15-1.35), eosinopenia (0 × 10^9^/L; RI: 0.1-1.25) and reticulocytosis (112 × 10^9^/L; RI: < 95) with normal hematocrit. Serum biochemistry identified hypoalbuminemia (19 g/L; RI: 24-38; 1.9 g/dL; RI: 2.4-3.8), low serum creatinine concentration (24 μmol/L; RI: 44-132; 0.27 mg/dL; RI: 0.5-1.5), total hypocalcemia (1.92 mmol/L; RI 2.2-2.8) with normal ionized calcium concentration, hyponatremia (139 mmol/L; RI: 150-165), hypochloremia (108 mmol/L; RI: 112-129), and hyperlactatemia (2.4 mmol/L; RI: 0.5-2). Urinalysis indicated urine specific gravity of 1.020 (RI: > 1.030), pH 5.5 (RI: 6.5-7.5), mild pyuria, and hematuria (9 cells/μL each; RI < 5). Abdominal ultrasonography identified moderate circumferential colonic wall thickening, intramural nodular colonic wall lesions, hyperechoic intramural foci, and moderate colic and iliac lymphadenomegaly. In addition, bilateral pinpoint nephroliths and numerous irregular-shaped urinary bladder calculi were noted. Mild rectal stenosis and moderate stenosis of the descending colon, the latter preventing easy passage of the endoscope, were identified on colonoscopy and positive contrast colonogram (Figure 1). A third stenotic area aboral to the ileocolic junction was suspected on contrast radiographs and confirmed by exploratory laparotomy. The ileo-ceco-colic junction and adjacent stenotic bowel were resected, and full thickness biopsy samples were obtained from gastric and small intestinal walls. Cystoliths were surgically removed and identified as calcium oxalate. Histopathology of ileum and colon indicated severe mixed inflammation and numerous round algal sporangia within macrophages were observed (Figure 2). No algae were found in gastric and duodenal biopsy samples. Algae were cultured on Sabouraud’s dextrose agar with chloramphenicol (SABC) from a rectal scrape and were speciated as either *P. bovis or P. ciferrii* by microscopic and biochemical analyses. The infecting species was definitively identified as *P. bovis* by PCR amplification and sequencing of the 28S LSU rDNA locus from DNA extracted from formalin-fixed, paraffin-embedded (FFPE) tissue^4^ (Table S1). Antimicrobial susceptibility testing determined minimal inhibitory concentrations (MIC) of commonly used drugs against *Prototheca* spp. (Table 1).

**Table 1:** Antimicrobial susceptibility data for 4 *P. Bovis* isolates.

	Dog 1	Dog 2	Dog 3	Dog 3
**Time of sampling**	At diagnosis	19 months into treatment	12 months into treatment	12 months into treatment
**Type of sample**	Rectal scrape	Rectal scrape	Feces	Spinal fluid
**Anidulafungin**	>8	N/A	>8	>8
**Micafungin**	>8	N/A	>8	>8
**Caspofungin**	8	N/A	N/A	>8
**5-Flucytosine**	>64	N/A	>64	>64
**Posaconazole**	0.25	2	1	>8
**Voriconazole**	2	2	2	>8
**Itraconazole**	0.25	1	1	>16
**Fluconazole**	>128	>256	>256	>128
**Amphotericin B**	0.25	1	2	2

Abbreviation: N/A = not available.

After surgery, signs of colitis and weight loss worsened. After 4 weeks treatment with posaconazole (Noxafil, 40 mg/mL suspension, Merck, Sharp & Dohme, Australia; 8 mg/kg/day PO), AMB (Fungizone R, Bristol-Myers Squibb, Canada; 0.7 mg/kg twice weekly as SC infusion in 400 mL of 0.45% NaCl and 2.5% dextrose), and dietary fiber (Psyllium husk) were commenced. After 12 administrations, AMB was discontinued because of progressive acute kidney injury. Signs of colitis started to improve after 1 week of treatment. After 2 months, the dog had regained normal appetite and BCS was 4/9. Persisting ultrasonographic colonic, colic, and iliac lymphadenopathy were noted after 12, 22, and 30 months of treatment. Repeat rectal scrape cytology and culture at 6 months showed persistent organisms and growth of algae, respectively. Both cytology and culture were negative after 22 months and remained negative after 30 months of treatment. Serum biochemical testing during treatment identified mild to moderate increases of liver enzyme activities, with no evidence of liver function deficit (Table S2). The dog was alive after 45 months on continuous treatment with posaconazole and with infrequent mild diarrhea. The trough serum posaconazole concentration after 28 months of treatment was 9.8 mg/L (39× the minimum inhibitory concentration [MIC] of the infecting algae).

### Case 2

A 2-year-old spayed female Shih Tzu was presented with a 6-month history of mixed-bowel diarrhea and 20% weight loss. Initial examination indicated a BCS of 4/9, mildly tense abdomen and mucoid feces with fresh blood. Blood tests showed monocytosis (1.95 × 10^9^/L; RI: 0.15-1.35), eosinopenia (0 × 10^9^/L; RI: 0.1-1.25), reticulocytosis (104 × 10^9^/L; RI: < 95) with normal hematocrit, low urea concentration (2.8 mmol/L; RI: 3.6-10; 7.8 mg/dL; RI: 10-28), hypoalbuminemia (21 g/L; RI: 24-38; 2.1 g/dL; RI: 2.4-3.8), and hyperglobulinemia (48 g/L; RI: 28-44; 4.8 g/dL; RI: 2.8-4.4). Abdominal ultrasonography identified a diffusely thickened colonic wall with prominent mucosal and submucosal layers and moderate colic and ileocolic lymphadenomegaly. Fine-needle aspirates of colic lymph nodes identified lymphoid hyperplasia, mixed inflammation, and occasional algae. Algae cultured on SABC from a rectal scrape were speciated as *P. bovis* by PCR and DNA sequencing of the cytochrome b (*cytb*) gene^5^ (Table S1).

Treatment with posaconazole suspension (8 mg/kg PO q24h) and dietary fiber (Psyllium husk) were commenced. Diarrhea had resolved after 3 months of treatment and the dog had gained body weight prompting discontinuation of dietary fiber. Mixed-bowel diarrhea with frequent hematochezia recurred and intermittent hyporexia developed; both failed to improve with reintroduction of fiber. Repeat examinations at 3, 9, and 21 months of treatment indicated static changes in the colonic wall on ultrasonography and algal growth on culture from rectal scrapes. Because of persistence of active colonic protothecosis, antimicrobial susceptibility testing was performed from a repeat algal culture after 19 months of treatment (Table 1). Trough serum posaconazole concentrations were 2× the MIC (4 mg/L). An extended-release formulation of the drug (Noxafil, 100 mg modified release tablets, Merck, Sharp & Dohme, Australia) was prescribed to improve absorption, but the dog refused to accept the tablets. The dose of posaconazole suspension therefore was arbitrarily increased to 12 mg/kg PO q24h. Improved fecal quality and decreased frequency of hematochezia were reported 4 weeks later. Monitoring of liver enzyme activities during treatment indicated a transient mild increase in alanine aminotransferase activity (Table S2). After 39 months of treatment, the dog had daily soft feces and occasional hematochezia but had no other clinical signs.

### Case 3

A 7-year-old spayed female Staffordshire Bull terrier cross was referred for 6 months of watery diarrhea with occasional fresh blood and mucus, tenesmus, hyporexia, and 35% weight loss. On initial examination, a BCS of 3/9 was noted. Blood tests identified neutrophilia (18.4 × 10^9^/L; RI: 6-14) with left shift, monocytosis (1.1 × 10^9^/L; RI: 0.2-1.0), eosinopenia (0 × 10^9^/L; RI: 0.1-1.2), hyponatremia (130 mmol/L; RI: 140-155) and hypochloremia (92 mmol/L; RI: 100-120). Abdominal ultrasonography indicated severe thickening of the transverse and ascending colon, moderate segmental thickening of the jejunum and distal ileum, focal pyloric wall thickening, and mesenteric lymphadenomegaly. Gastrointestinal endoscopy identified focal gastric thickening, erythema and white raised nodules in the duodenum, thickening of the ileocolic valve, and a colonic mass. Histopathologic examination of endoscopic biopsy samples showed pyloric fibrosis, plasmacytic duodenal inflammation, and mixed inflammation with moderate numbers of algae in cecal and colonic walls. The colonic mass was identified as the muscularis layer infiltrated by numerous protothecal organisms and mixed inflammatory cells. No algae were noted in smears from mesenteric lymph node aspirates.

**Figure 3:**
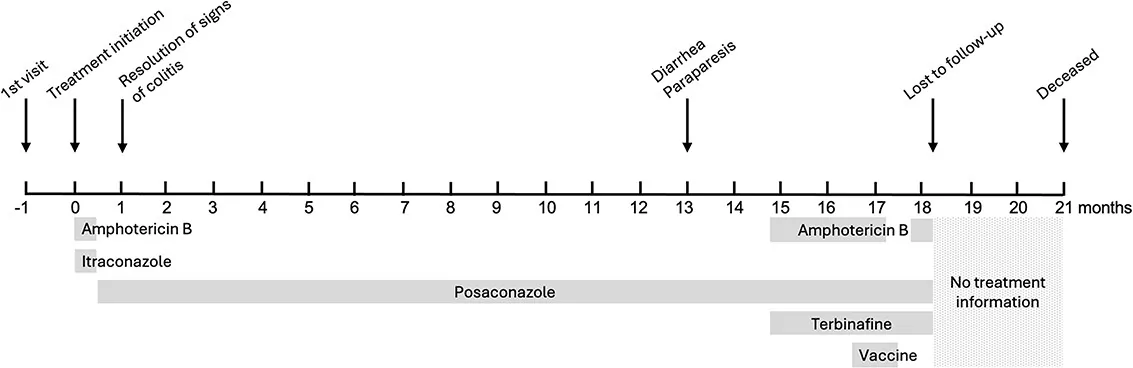
Timeline of antifungal treatments of dog 3. Relevant clinical details are indicated above the timeline. Duration of antifungal treatment is equivalent to the gray bars underneath the timeline. In this dog, an autologous vaccine was administered in addition to antifungal drugs.

The dog received itraconazole (Sporanox, 100 mg tablets, Janssen-Cilag Australia; 10 mg/kg PO q24h) and AMB (0.7 mg/kg SC infusion twice weekly as described for case 1; Figure 3). Sterile abscessation at the infusion site prompted discontinuation of the drug after 2 weeks. At the same time, itraconazole was discontinued and extended release posaconazole (5 mg/kg PO q24h) was commenced because of its ability to achieve high serum concentrations.^6^ Fecal consistency and BCS normalized within the first month of treatment. For 12 months, the dog remained free of clinical signs. Thirteen months into treatment, paraparesis, hind limb weakness, and frequent diarrhea developed. Cerebrospinal fluid analysis showed mixed cell pleocytosis and protothecal organisms. Algae were cultured on SABC and identified as *P. bovis* or *ciferrii* by matrix-assisted laser desorption/ionization time-of-flight mass spectrometry. Simultaneously, algae were cultured from a rectal swab. Species identity was resolved upon sequencing of the 28S LSU rDNA region, amplified by PCR from FFPE-derived DNA from previous intestinal biopsy samples^4^ (Table S1) and the strain subsequently identified as *P. bovis*. MIC were determined for both isolates (Table 1). Over the course of the next 3 months, AMB (dosed as described above), terbinafine (30 mg/kg PO q12h) and extended release posaconazole were administered. Additional immunotherapy using the *P. bovis* isolate from this patient was commenced as described previously for the treatment of pythiosis in dogs.^7^ The dog received 3 doses of a killed adjuvanted *Prototheca* vaccine produced from the dog’s fecal isolate (Treidlia Biovet, New South Wales, Australia). Diarrhea and weakness improved but paraparesis persisted and fecal incontinence developed. Infrequently monitored liver enzyme activities remained within reference intervals during treatment. The dog was euthanized 21 months after commencing treatment.

## Discussion

To date, approximately 130 cases of protothecosis have been described in dogs in the veterinary literature.^1^^,^^4^^,^^8^ Ninety-nine dogs were presented with signs of systemic (*n* = 86) or enteric (*n* = 13) infection and 30 of these dogs received antifungal treatment (Table S3). Extrapolated survival times were 5.1 months (range, 0.1-56 months) for systemic and 5.2 months (range, 1-12 months) for enteric infection with all but 3 dogs dying as a consequence of protothecosis. Surviving dogs received a combination of AMB IV and itraconazole PO (follow-up 2 and 12 months) or nystatin PO (follow-up 3 months).^2^^,^^9^^,^^10^ In contrast, the 3 dogs in our case report survived for > 20 months after commencing antifungal treatment and 2 dogs were alive for > 3 years without signs of disseminated disease.

A possible cause for the prolonged survival in our cases is the use of posaconazole as the mainstay of treatment or as the sole agent. Posaconazole is a broad spectrum triazole antifungal agent with a large volume of distribution and accumulation in various tissues.^11^^,^^12^ Importantly, the drug is exceedingly well tolerated in dogs, allowing administration of high doses to achieve high serum trough concentrations.^6^^,^^13^ To our knowledge, the use of posaconazole has not been described for treatment of localized protothecosis in dogs and only once for treatment of disseminated disease resulting in robust clinical improvement; discontinuation of the drug after 2 weeks and commencement of itraconazole was associated with progressive disease and euthanasia.^14^

Posaconazole has been used successfully in various fungal diseases of dogs including disseminated aspergillosis,^15^ penicilliosis,^6^  *Chrysosporium* spp. infections,^16^ refractory sino-nasal aspergillosis,^17^ and coccidiomycosis.^13^ Given its efficacy against fungal infections, administration of posaconazole also may have useful efficacy for management of protothecosis in dogs despite taxonomical and biochemical differences between fungi and algae.

Susceptibility of the protothecal organisms to posaconazole and AMB, if used, may have further contributed to the long survival times in our cases. The lowest MICs were noted for AMB, posaconazole, itraconazole, and voriconazole (Table 1). This observation is comparable to previous studies of 148 isolates of *P. bovis* and *P. zopfii* (genotype 2) that reported the lowest MICs for AMB (0.19), posaconazole (0.5), voriconazole (0.5), and itraconazole (1.5; Table S4). Whereas data regarding the correlation of in vitro and in vivo susceptibility of *Prototheca* spp. are lacking, the best response to treatment in our study was seen for the isolates with the lowest MICs for posaconazole and AMB (dog 1), where algae were no longer detectable after 22 months of treatment. However, the MIC’s reported for the isolates from the other 2 dogs were measured after 19 (dog 2) and 12 months (dog 3) of treatment, and it is possible or indeed likely that mutational resistance developed in these dogs, resulting in progressively higher MICs. Moreover, dog 1 underwent surgical reduction of the algae burden. As such, it remains speculative whether treatment success in this dog was attributable to the favorable susceptibility of the infecting algae alone.

Treatment of protothecosis before dissemination is another possible factor for prolonged survival. Localized algal infection of tissues that are readily penetrated by antifungal drugs is thought to carry a better prognosis.^1^ In all 3 dogs in our case series, signs of widespread dissemination were absent at the time treatment was initiated suggesting that the disease was largely confined to the gastrointestinal tract. However, diagnostic investigations of organs typically affected by disseminated protothecosis such as the CNS and eyes had not been performed before treatment because supportive signs were absent. Subclinical dissemination before starting treatment therefore cannot be excluded, especially in dog 3 where algae were identified in the cerebrospinal fluid after the first year of antifungal treatment.

The clinical course of protothecosis varied among the 3 dogs in our case series. This variation may be associated with antimicrobial susceptibility of individual algae isolates as explained above. Moreover, absorption of posaconazole may have been variable as indicated by the difference in trough concentrations between dog 1 (9.8 mg/L) and 2 (4.0 mg/L). Serum trough concentrations were not measured in dog 3. Decreased bioavailability of posaconazole suspension has been described in humans if the drug is administered without food or if diarrhea is present.^18^^,^^19^ Dog 2 experienced ongoing diarrhea, possibly resulting in a lower trough concentration although variable bioavailability alone may have accounted for the difference as previously shown in dogs.^20^

Finally, antimicrobial drug resistance, both instrinsic and acquired, may have influenced clinical outcome. Whereas antimicrobial resistance is well described for various infectious organisms,^21^ comparable data for algae are lacking. Resistance may have developed in dog 3 that experienced CNS dissemination while undergoing treatment with posaconazole. It was thus of great interest that algae isolated from cerebrospinal fluid had > 8 times higher MIC for posaconazole than algae simultaneously cultured from the colon. Because posaconazole does not cross the blood–brain barrier in the absence of inflammation, a transient entry of the drug into the CNS during a period of inflammation resulting in an inadequate duration of exposure may have facilitated development of resistance.

Administration of posaconazole was well tolerated in all of the dogs. Adverse effects of posaconazole in humans are primarily confined to hepatotoxicity.^11^^,^^12^ Hepatic dysfunction is reported infrequently and typically is readily reversible on drug discontinuation. In our case report, 2 dogs exhibited mild to moderate increases in hepatic enzyme activities, but no evidence of hepatic failure. These findings suggest that posaconazole can be a safe, efficacious, and affordable therapeutic option for the management of intestinal protothecosis in dogs.

## Supplementary Material

Supplementary_material_aalag170

## Off-label antimicrobial declaration

Authors declare no off-label use of antimicrobials.

## Institutional animal care and use committee or other approval declaration

Authors declare no institutional animal care and use committee or other approval was needed.

## Abbreviations

BCS: body condition score
CNS: central nervous system
MIC: minimal inhibitory concentrations
P: Prototheca
RI: reference interval
SABC: Sabouraud’s dextrose agar with chloramphenicol
spp: several species
