# Supplementary material for: Prolonged survival of 3 dogs with Prototheca bovis infection treated with posaconazole
Source: J Vet Intern Med. 2026 Aug 24;40(4):aalag170. doi: 10.1093/jvimsj/aalag170 (PMC13502398; doi:10.1093/jvimsj/aalag170)
Supplement: Supplementary file 1 [file supplementary_material_aalag170.zip › Supplemental Table 3.docx]

**Supplemental Table 3. Published reports of dogs with enteric or disseminated protothecosis receiving treatment with antifungal drugs.**

| **No.** | **Signalment** | **Infecting species** | **Initially affected site of infection^a^** | **Subsequent sites of infection^b^** | **Dissemination at start of antifungal treatment^c^** | **Anatomic sites of infection^d^** | **Antifungal treatment** | **Survival (months)^e^** | **Outcome** | **Ref.** |
| --- | --- | --- | --- | --- | --- | --- | --- | --- | --- | --- |
| 1 | Cross  7 years | *P. zopfii*^f^ | Eyes | Kidneys | Yes^g^ | Eyes, CNS, heart, kidneys, aorta | Amphotericin B IV Amphotericin B intravitreal  Ketoconazole PO | 4 | Improvement of ocular signs with high dose amphotericin B  Progressive ocular disease with lower doses of amphotericin B  Stable to improved ocular disease following intravitreal amphotericin B  Died whilst receiving ketokonazole | ^1^ |
| 2 | English Cocker Spaniel  8 years  FN | *P. zopfii* | GIT | Eyes | Yes | GIT, eyes, CNS, heart, kidneys, liver | Ketokonazole PO | 0.5 | Improvement of colitis whilst receiving antifungal treatment  Euthanasia after development of renal failure | ^2^ |
| 3 | Cross  5 years  FN | N/A | Eyes | GIT, CNS | No | GIT, eyes, CNS, heart, kidneys, liver, pancreas | Itraconazole PO | 10 | Progressive ocular disease requiring bilateral enucleation  No clinical signs for 9 months with ongoing treatment  Euthanasia after development of GIT and subsequently CNS signs | ^3^ |
| 4 | Giant Schnauzer  1 year  M | *P. zopfii* | GIT | Eyes, skin, lymph nodes | Yes | GIT, eyes, kidneys, lymph nodes, skin | Ketoconazole PO Amphotericin B IV | 2 | Resolution of colitis  Euthanasia after development of cutaneous signs and general decline | ^4^ |
| 5 | German Shepherd  3.5 years  MN | N/A | GIT | None | No | GIT (no algae detected in eyes, kidneys, liver, lungs) | Ketoconazole PO | 5 | Several months of clinical remission  Euthanasia after recurrence of colitis | ^5^ |
| 6 | German Shepherd  6 years  F | N/A | GIT | Eyes, CNS | Yes | GIT (no other tissues examined) | Ketoconazole PO  Nystatin PO | 1 | Stable ocular and neurological signs and improvement of signs of colitis  Died after decline | ^6^ |
| 7 | Cocker Spaniel  2 years  MN | N/A | GIT | None | No | GIT (no other tissues examined) | Amphotericin B IV  Clotrimazole enema  Amphotericin B enema  Itraconazole PO  Ketoconazole PO | 12 | Several months of clinical remission  Relapse of colitis after discontinuation of drugs  Regain of remission  Died after recurrence colitis following second discontinuation of drugs | ^7^ |
| 8 | Cross  8 years  FN | *P. zopfii* | Eyes | CNS, GIT | Yes | GIT, eyes, CNS, kidneys | Itraconazole PO | 3 days | Euthanasia due to lack of improvement | ^8^ |
| 9 | Boxer  2 years  FE | *P. wickerhamii* | GIT | None | No | GIT, kidneys, mesenteric lymph nodes | Amphotericin B SC  Itraconazole PO | 9 | Several months of clinical remission  Euthanasia after relapse of colitis despite antifungal treatment | ^9^ |
| 10 | Cross  6 years  FN | N/A | GIT | Eyes, CNS | Yes | GIT, eyes | Amphotericin B (route of administration unknown)  Itraconazole PO | 12 | Alive  No information regarding clinical status available | ^9^ |
| 11 | Maltese  4 years  MN | N/A | GIT | None | No | GIT (no other tissues examined) | Amphotericin B (route of administration unknown) | 4 | No details available regarding clinical course  Euthanasia | ^9^ |
| 12 | Cross  1.5 years  M | N/A | GIT | None | No | GIT (no other tissues examined) | Ketoconazole PO | 4 | Mild improvement of colitis for a few weeks  Euthanasia due to rapid decline despite antifungal treatment | ^10^ |
| 13 | Cross  8 years  ME | *P. zopfii* | GIT | None | No | GIT (no other tissues examined) | Nystatin PO | 3 | Alive  Resolution of colitis and rectal scrapes negative for algae | ^11^ |
| 14 | Cross  3 years  FN | *P. zopfii* | GIT | CNS | Yes | GIT, CNS (no other tissues examined) | Itraconazole PO  Amphotericin B intrathecally  Amphotericin B IV | 1 | Mild improvement of clinical signs for a few weeks  Euthanasia after decline despite antifungal treatment | ^12^ |
| 15 | Labrador Retriever  5 years  F | *P. zopfii* | CNS | Eyes | No | CNS (no evidence for algae in other organs on necropsy) | Ketoconazole PO | 6 | Development of ocular signs after 1 month of treatment  Died following acute neurological decline | ^13^ |
| 16 | Rough Collie  2 years  FE | *P. zopfii* | CNS | Eyes, GIT | Yes | GIT, eyes, CNS, heart, kidneys | Itraconazole PO  Nystatin PO | 5 days | Euthanasia following decline despite treatment | ^14^ |
| 17 | Labrador Retriever  6 years  F | N/A | Eyes | CNS | Yes | GIT, eyes, CNS, heart, kidneys, thyroid and parathyroid glands, vertebrae, skeletal muscle, subcutaneous fat | Voriconazole PO | 5 days | Euthanasia following decline despite treatment | ^15^ |
| 18 | Rhodesian ridgeback  6 years  FN | *P. zopfii* (genotype 2) | GIT | Eyes, lymph nodes | Yes | GIT, peripheral lymph nodes | Itraconazole PO | 1 | Euthanasia due to decline despite antifungal treatment | ^16^ |
| 19 | Rough Collie  2 years  FE | *P. wickerhamii* | Eyes, CNS, respiratory tract | GIT, skin, tongue | Yes | Eyes, skin (no other tissues examined) | Itraconazole PO  Terbinafine PO Clotrimazole topically | 2 | No improvement of neurological, ocular and GIT signs with itraconazole and development of cutaneous and tongue lesions prompting discontinuation of the drug  Progressive mucocutaneous lesions with terbinafine and topical clotrimazole  Died whilst receiving antifungal treatment | ^17^ |
| 20 | Cross  11 years  FN | *P. zopfii* | GIT | Eyes | No | GIT (no other tissues examined) | Itraconazole PO | 1.5 | Euthanasia due to lack of improvement of colitis and onset of signs suggestive of dissemination despite 11antifungal treatment | ^18^ |
| 21 | Maremma Sheepdog  11 years  F | *P. zopfii* | GIT | Eyes | Yes | GIT, kidney, liver, pancreas, mesenteric lymph nodes, skeletal muscles abdomen, SC tissue, peritoneum | Itraconazole PO | 2 | No improvement of clinical signs despite antifungal treatment  Died | ^18^ |
| 22 | Boxer  5 years  M | *P. zopfii* | GIT | None | No | GIT (no other tissues examined) | Itraconazole PO | 2 | A few weeks of improvement of colitis  Died whilst receiving antifungal treatment | ^18^ |
| 23 | Boxer  6 years  F | *P. zopfii* | GIT | CNS | No | GIT (no other tissues examined) | Itraconazole PO | 5 | Several months of improvement of colitis  Euthanasia due to onset of neurological signs whilst receiving antifungal treatment | ^18^ |
| 24 | Hungarian Vizla  2 years  FN | N/A | GIT | Eyes, CNS | Yes | GIT, eyes, CNS, kidney, thyroid gland, abdominal lymph nodes | Itraconazole PO  Posaconazole PO | 1 | Worsening of clinical signs whilst receiving itraconazole Marked improvement with posaconazole  Rapid decline and subsequent euthanasia after stopping posaconazole and recommencing itraconazole | ^19^ |
| 25 | French Bulldog  3 years  F | *P. zopfii* | GIT | Eyes | Yes | GIT (no other tissues examined) | Itraconazole PO  Amphotericin B IV | 5 | Improvement of clinical signs and eventually resolution of signs of colitis  Relapse and death 2 weeks after stopping antifungal medications | ^20^ |
| 26 | Cross  4 years  ME | *P. zopfii* (genotype 1) | Skin | Eyes, GIT | Yes | Skin (no other tissues examined) | Amphotericin B IV  Itraconazole PO | 2 | Improvement of cutaneous lesions, no information regarding GIT and ocular signs  Lost to follow up | ^21^ |
| 27 | Scottish Shepherd  4 years  ME | *P. zopfii* (genotype 2) | GIT | Eyes, CNS | Yes | GIT, CNS, kidneys (urine culture revealed algal growth) | Itraconazole PO | 0.75 | Mild improvement of neurological signs followed by worsening whilst receiving antifungal treatment  Euthanasia | ^22^ |
| 28 | Cross  9 years  FN | N/A | GIT | Eyes | No | GIT, eyes, (no other tissues examined) | Itraconazole PO | 1 | Mild improvement of clinical signs  Euthanasia | ^23^ |
| 29 | Scottish Collie  3 years  FN | N/A | GIT | CNS | Yes | GIT (no other tissues examined) | Nystatin PO | 0.25^h^ | Euthanasia due to lack of improvement with antifungal treatment | ^24^ |
| 30 | Cross, 5 years, FN | *P. bovis* | Eyes | CNS | Yes | GIT, left eye and kidneys at initial diagnosis; GIT, right eye, CNS, heart, kidneys, thyroid gland, lymph nodes, arteries at necropsy | Itraconazole PO, liposomal amphotericin B IV, terbinafine PO, doxycycline PO, nystatin PO, isavuconazole PO | 56 | Remission following initial treatment with itraconazole and amphotericin B, relapse after 4 years with ongoing itraconazole, euthanasia after failure to improve long-term | ^25^ |

N/A: not admitted; M: male; ME: male entire; MN: male neutered; F: female; FE: female entire; FN: female neutered; CNS: central nervous system; GIT: gastrointestinal tract; PO: per os; IV: intravenous; SC: subcutaneous.

^a^Based on presenting clinical signs

^b^Based on developing clinical signs

^c^Presence of dissemination determined by either clinical signs pertaining to various organ systems typically affected by protothecosis (CNS, eyes, skin) or detection of algae in organs on cytology, histopathology or fungal culture

^d^Site included if algae were identified on cytology or histopathology

^e^Survival time after starting antifungal treatment

^f^*P. zopfii* refers to either *P. bovis* or *P. ciferrii*

^9^Blood culture grew *Prototheca* spp.

^h^Survival time estimated as 1 week as per personal communication with P. Mandigers

**References**

1.	Moore FM, Schmidt GM, Desai D, Chandler FW. Unsuccessful treatment of disseminated protothecosis in a dog. J Am Vet Med Assoc. 1985 Apr 1;186(7):705–8.

2.	Meehan C, Pollock J. *Prototheca* *zopfii* infection in a Cocker Spaniel. Australian Veterinary Practitioner. 1996 Sep;26(3):146–7.

3.	Schultze, Ring, Morgan, Patton. Clinical, cytologic and histopathologic manifestations of protothecosis in two dogs. Vet Ophthalmol. 1998 Jan 5;1(4):239–43.

4.	Allgoewer I, Ehrlein J, Goebel T, Hermanns W, Leibold W, Schaeffer E, et al. Disseminated protothecosis in a Giant Schnauzer. Kleintierpraxis. 1998;43(5):375–91.

5.	Rallis T, Tontis D, Adamama‐Moraitou K, Mylonakis M, Papazoglou L. Protothecal colitis in a German Shepherd Dog. Aust Vet J. 2002 Jul 10;80(7):406–8.

6.	Birke L. Protothecosis in a German Shepherd Dog. Praktischer Tierarzt. 2002;83(11):952–7.

7.	Strunck E, Billups L, Avgeris S. Canine protothecosis. Compendium. 2004 Feb;26:96–102.

8.	Pressler BM, Gookin JL, Sykes JE, Wolf AM, Vaden SL. Urinary tract manifestations of protothecosis in dogs. J Vet Intern Med. 2005;19(1):115.

9.	Stenner VJ, MacKay B, King T, Barrs VRD, Irwin P, Abraham L, et al. Protothecosis in 17 Australian dogs and a review of the canine literature. Med Mycol. 2007 Jan;45(3):249–66.

10.	Sapierzyński R, Jaworska O. Protothecosis as a cause of chronic diarrhoea in a dog. Pol J Vet Sci. 2008;11(3):225–9.

11.	Ribeiro MG, Rodrigues de Farias M, Roesler U, Roth K, Rodigheri SM, Ostrowsky MA, et al. Phenotypic and genotypic characterization of *Prototheca* *zopfii* in a dog with enteric signs. Res Vet Sci. 2009 Dec;87(3):479–81.

12.	Young M, Bush W, Sanchez M, Gavin P, Williams M. Serial MRI and CSF analysis in a dog treated with intrathecal amphotericin B for protothecosis. J Am Anim Hosp Assoc. 2012 Mar 1;48(2):125–31.

13.	Font C, Mascort J, Márquez M, Esteban C, Sánchez D, Durall N, et al. Paraparesis as initial manifestation of a *Prototheca zopfii* infection in a dog. Journal of Small Animal Practice. 2014 May 7;55(5):283–6.

14.	Vince AR, Pinard C, Ogilvie AT, Tan EO, Abrams-Ogg ACG. Protothecosis in a dog. Can Vet J. 2014 Oct;55(10):950–4.

15.	Manino PM, Oliveira F, Ficken M, Swinford A, Burney D. Disseminated protothecosis associated with diskospondylitis in a dog. J Am Anim Hosp Assoc. 2014 Nov 1;50(6):429–35.

16.	Beribè F, Miglio A, Cassarani MP, Magi G, Passamonti F, Laus F, et al. What is your diagnosis? Systemic lymphadenopathy and blindness in a dog from Italy. Vet Clin Pathol. 2014 Dec 10;43(4):605–6.

17.	Milley CD, Labelle P, Tolar E, Griffin J, Reeder C. *Prototheca* *wickerhamii* in a dog with cutaneous, ocular and systemic manifestations and treatment with terbinafine. Vet Rec Case Rep. 2015 Jan 7;3(1).

18.	Bottero E, Mercuriali E, Abramo F, Dedola B, Martella V, Zini E. Fatal protothecosis in four dogs with large bowel disease in Italy. Wien Tierarztl Monatsschr. 2016 Dec 14;103:17–21.

19.	Enders B, Olby N, Mariani CL. Use of posaconazole for treatment of disseminated protothecosis in a dog. Vet Rec Case Rep. 2017 Feb 2;4(2).

20.	Spampinato MF, Kujman S, Cantón J, Daglio C, Catena M. Prototecosis canina: primer reporte en Argentina. Revista Veterinaria. 2017 Nov 1;28(2):168–71.

21.	Silveira CS, Cesar D, Keating MK, DeLeon-Carnes M, Armién AG, Luhers M, et al. A case of *Prototheca* *zopfii* genotype 1 infection in a dog (*Canis* *lupus* *familiaris*). Mycopathologia. 2018 Oct 6;183(5):853–8.

22.	Cocchetto A, Briola C, Furlanello T, Danesi P, Cirla A, Menchetti M. 3‐T MRI of protothecosis encephalic lesions in a Scottish Shepherd Dog. Vet Rec Case Rep. 2020 Sep 23;8(3).

23.	Price P, Klobukowska H, Castillo-Alcala F, Foxwell J, Orbell G, Brown S, et al. Protothecosis in four dogs in New Zealand. N Z Vet J. 2023 Nov 2;71(6):321–8.

24.	Bontekoning I, Maes SMJ, Mandigers PJJ. A dog with protothecosis in the Netherlands. Vet Rec Case Rep. 2024 Mar 16;12(1).

25.	Landfield BG, Appleman EH, Daverio H, Guarino C, Moore ME, Gioia G. Extended Survival in a Dog With Disseminated Protothecosis. J Vet Intern Med. 2025 Sep 27;39(5).
