# Supplementary material for: Prolonged survival of 3 dogs with Prototheca bovis infection treated with posaconazole
Source: J Vet Intern Med. 2026 Aug 24;40(4):aalag170. doi: 10.1093/jvimsj/aalag170 (PMC13502398; doi:10.1093/jvimsj/aalag170)
Supplement: Supplementary file 1 [file supplementary_material_aalag170.zip › Supplemental Table 4.docx]

**Supplemental Table 4. *In vitro* susceptibility of *Prototheca* spp^a^ isolates to antifungal drugs shown as minimum inhibitory concentrations^b^.**

| **Origin of isolates** | **Species** | **Number of isolates** | **AMB** | **MCZ** | **FLU** | **KTZ** | **ITZ** | **PCZ** | **VRZ** | **RVZ** | **EFZ** | **ICZ** | **5-FC** | **CAS** | **MIC** | **ANI** | **NYT** | **Ref.** |
| --- | --- | --- | --- | --- | --- | --- | --- | --- | --- | --- | --- | --- | --- | --- | --- | --- | --- | --- |
| Bovine mastitis | *P. zopfii* (genotype 2) | 10 | **1.31^GM^**  (0.25-4) |  |  |  | **>32^GM^** |  |  |  |  |  |  |  |  |  |  | ^1^ |
| Bovine mastitis | *P. zopfii* (genotype 2) | 113 | **1.5^GM^**  (0.19-32) |  | **>256^GM^** | **>32^GM^**  (0.75->32) | **>32^GM^**  (1.5->32) | **>32^GM^**  (0.5->32) | **>32^GM^**  (0.5->32) |  |  |  | **>32^GM^** | **>32^GM^** | **>32^GM^** | **>32^GM^** |  | ^2^ |
| Infected humans | *P. bovis* | 3 | **0.5^M^**  (0.5-1) | **128^M^**  (32-128) | **128^M^**  (64-128) | **8^M^**  (4-32) | **32^M^**  (4->32) | **4^M^**  (1-32) | **8^M^**  (4-16) | **8^M^**  (0.5-1) | **0.25^M^**  (0.125-0.5) |  |  |  |  |  |  | ^3^ |
| Canine protothecosis | *P. bovis* | 21 | **2^M^**  (0.25-8) |  | **64^M^**  (16-128) | **16^M^**  (2-32) | **32^M^**  (2-128) |  |  | **0.5^M^**  (0.063-2) | **0.25^M^**  (0.031-0.5) |  |  |  |  |  |  | ^4^ |
| Canine protothecosis | *P. bovis*^c^ | 1 | 4 |  | >256 |  | >16 | >8 | >8 |  |  | 1 | >64 | >8 | >8 | >8 | 8 | ^5^ |

N/A: not admitted; AMB, amphotericin B; MCZ, miconazole; FLU, fluconazole; KTZ, ketoconazole; ITZ, itraconazole; PCZ, posaconazole; VRZ, voriconazole; RVZ, ravuconazole; EFZ, efinaconazole; ICZ, isavuconazole; 5-FC, 5-flucytosin; CAS, caspafungin; MIC, micafungin; ANI, anidulafungin; NYT, nystatin

^a^Isolates included were *P. zopfii* (genotype 2) and *P. bovis*

^b^If ≥2 isolates were investigated, MIC is reported as **median^M^** (range) or **geometric mean^GM^** (range)

^c^Susceptibility testing performed almost 4 years into treatment with amphotericin B and itraconazole

**References**

1. Sobukawa H, Kano R, Ito T, Onozaki M, Makimura K, Hasegawa A, et al. *In* *vitro* susceptibility of *Prototheca* *zopfii*
 genotypes 1 and 2. Med Mycol. 2011 Feb;49(2):222–4.

2.	Jagielski T, Buzzini P, Lassa H, Malinowski E, Branda E, Turchetti B, et al. Multicentre Etest evaluation of *in* *vitro* activity of conventional antifungal drugs against European bovine mastitis *Prototheca* spp. isolates. Journal of Antimicrobial Chemotherapy. 2012 Aug 1;67(8):1945–7.

3.	Proskurnicka A, Żupnik K, Bakuła Z, Iskra M, Rösler U, Jagielski T. Drug susceptibility profiling of *Prototheca* species isolated from cases of human protothecosis. Antimicrob Agents Chemother. 2023 Apr 18;67(4).

4.	Proskurnicka A, Iskra M, Wronka S, Bakuła Z, Danesi P, de Farias MR, et al. Genotyping and drug susceptibility profiling of *Prototheca* sp. strains isolated from cases of protothecosis in dogs. J Vet Intern Med. 2025 Jan 20;39(1).

5.	Landfield BG, Appleman EH, Daverio H, Guarino C, Moore ME, Gioia G. Extended Survival in a Dog With Disseminated Protothecosis. J Vet Intern Med. 2025 Sep 27;39(5).
