# Supplementary material for: Prolonged survival of 3 dogs with Prototheca bovis infection treated with posaconazole
Source: J Vet Intern Med. 2026 Aug 24;40(4):aalag170. doi: 10.1093/jvimsj/aalag170 (PMC13502398; doi:10.1093/jvimsj/aalag170)
Supplement: Supplementary file 1 [file supplementary_material_aalag170.zip › Supplemental Table 1.docx]

**Supplemental Table 1. Methodology of molecular species identification.**

|  | **Case 1 and 3** | **Case 2** |
| --- | --- | --- |
| **Material** | Formalin-fixed, paraffin-embedded tissue | Algae culture from a rectal scrape |
| **DNA isolation** | DNA isolation was done with the Cobas DNA Sample Preparation Kit (Roche) according to the manufacturer’s instructions with three modifications: (i) one to five 5-µm tissue sections were incubated with xylene for 15 min at 37°C; (ii) following ethanol washing, samples were incubated at 56 °C for 2 hrs; and (iii) the resulting pellet was digested in TLB buffer (Roche) with proteinase K at 56 °C for 2 hrs. | Genomic DNA was extracted from a fresh culture using the GeneMATRIX Environmental DNA & RNA Purification Kit (EURx, Poland). |
| **DNA amplification and sequencing** | The extracted DNA was amplified and sequenced using NL-1 (5′-GCATATCAATAAGCGGAGGAAAAG-3′) and NL-4 (5′-GGTCCGTGTTTCAAGACGG-3′) primers targeting the 28S large ribosomal subunit (LSU) rDNA region.^1^ | PCR amplification and DNA sequencing were performed with cytb-F1 (5′-GYGTWGAACAYATTATGAGAG-3′) and cytb-R2 (5′-WACCCATAARAARTACCATTCWGG-3′) primers.^1^ |

**References**

1.	Jagielski T, Gawor J, Bakuła Z, Decewicz P, Maciszewski K, Karnkowska A. *cytb* as a New Genetic Marker for Differentiation of Prototheca Species. J Clin Microbiol. 2018 Oct;56(10). doi:10.1128/JCM.00584-18
