# Supplementary material for: Prolonged survival of 3 dogs with Prototheca bovis infection treated with posaconazole
Source: J Vet Intern Med. 2026 Aug 24;40(4):aalag170. doi: 10.1093/jvimsj/aalag170 (PMC13502398; doi:10.1093/jvimsj/aalag170)
Supplement: Supplementary file 1 [file supplementary_material_aalag170.zip › Supplemental Table 2.docx]

**Supplemental Table 2. Liver enzyme activities and liver function parameters at diagnosis and whilst undergoing treatment with posaconazole.**

|  | Dog 1 | |  |  |  |  |  |  | Dog 2 | |  | |  | |  |  | Dog 3 | |  |  | |
| --- | --- | --- | --- | --- | --- | --- | --- | --- | --- | --- | --- | --- | --- | --- | --- | --- | --- | --- | --- | --- | --- |
|  | Before treatment | | 8  mths | 10 mths | 20 mths | 28 mths | 42 mths |  | Before treatment | | 3 mths | 6 mths | | 19 mths | 29 mths |  | Before treatment | | 14 mths | 15 mths |  |
|  |  | RI |  |  |  |  |  | RI |  | RI |  |  | |  |  | RI |  | RI |  |  | RI |
| AST (U/L) | 58 | 1-60 | **78^a^** | 48 | **70** | **94** | **60** | 18-56 | 38 | 1-60 | **75** | 37 | | 47 | 42 | 18-56 | 30 | 1-80 | 24 | 11 | 21-52 |
| ALT (U/L) | 30 | 21-142 | **335** | **454** | **538** | **256** | **416** | 19-107 | 11 | 21-142 | **140** | 35 | | **18** | **13** | 19-107 | 21 | <80 | 29 | 14 | 18-89 |
| ALP (U/L) | 68 | 20-184 | 28 | 30 | 43 | 40 | 114 | 21-170 | 45 | 20-184 | 42 | 35 | | 34 | 39 | 21-170 | 38 | 1-120 | 104 | 58 | 11-129 |
| GGT (U/L) | <1 | 1-8 | 4 | 6 | 5 | 4 | **13** | 0-6 | 3 | 1-8 | 6 | 6 | | **7** | 4 | 0-6 | <5 | <6 | <5 | 5 | <7 |
| Urea (mmol/L) | 3.8 | 3.6-10 | 6.5 | 6.2 | 7.2 | 7.7 | 7.8 | 3.2-9.3 | **2.8** | 3.6-10 | 5.1 | 4.8 | | 4.7 | 7.3 | 3.2-9.3 | 3.6 | 2.5-9 | 3.4 | **2.5** | 3-9.8 |
| Glucose (mmol/L) | 4.6 | 3.6-6.8 | 4.6 | **2.9^b^** | 5.2 | 4.8 | 4.7 | 3.4-6.7 | 4.4 | 3.6-6.8 | 4.9 | 6.6 | | **1^b^** | 5.9 | 3.4-6.7 | 5.7 | 3.5-6.7 | 4.3 | **6.6** | 3.6-6.2 |
| Albumin (g/L) | **20** | 24-38 | 28 | 28 | **26** | 27 | **22** | 27-39 | **21** | 24-38 | 31 | 27 | | **23** | **24** | 27-39 | 32 | 22-36 | 28 | **26** | 28-44 |
| Cholesterol (mmol/L) | 6.5 | 3.3-6.9 | 7.1 | 6.5 | 6.5 | 6.3 | 6.2 | 3.5-10 | 5.5 | 3.3-6.9 | **11.4** | 7.9 | | 6 | 7.2 | 3.5-10 | 4.2 | 3.6-8.8 | **14.1** | **11.6** | 3.6-10 |
| Bilirubin (umol/L) | <2 | 2-17 | <2 | 1 | 1 | 3 | 2 | 0-4 | <2 | 2-17 | <2 | 1 | | 2 | <1 | 0-4 | 1 | <11 | 1 | 1 | <6 |

AST, aspartate transaminase; ALT, alanine transaminase; ALP, alkaline phosphatase; GGT, gamma-glutamyl transferase; mths, months; RI, reference interval

^a^Abnormal measurements highlighted in bold

^b^Glucose measured from clotted sample
